# Vaginal cuff dehiscence and bowel evisceration as a late-onset complication of vaginal vault prolapse: a case report

**DOI:** 10.1186/s13256-026-06214-5

**Published:** 2026-06-13

**Authors:** Carolin Schröder, Eva K. Egger, Jakob Friedemann Pantenburg, Jonas Dohmen, Laura Tascón Padrón, Lucia A. Otten, Alexander Mustea, Dominique Koensgen

**Affiliations:** 1 Department of Gynaecology and Gynaecological Oncology, University Hospital Bonn, Venusberg Campus 1, 53127 Bonn, Germany; 2 Department of Surgery, University Hospital Bonn, Venusberg Campus 1, 53127 Bonn, Germany

**Keywords:** Vaginal dehiscence, Vaginal evisceration, Vaginal hysterectomy

## Abstract

**Background:**

Vaginal cuff dehiscence (and evisceration, VCDE) after hysterectomy is a rare, but potentially serious complication. Current medical literature describes the most critical risk factors for VCDE after hysterectomy as surgical technique, use of thermal energy, early mechanical stress (especially sexual intercourse), infection, smoking, and obesity. There is currently no evidence regarding a potential association between VCDE and anti-HER2 therapy. We report a late-onset VCDE in a patient receiving combined antihormonal and anti-HER2 therapy, and discuss the hypothesis-generating question of a potential association, as well as the bowel-preserving surgical management.

**Case presentation:**

We report the case of an 81-year-old White woman with hormone receptor-positive breast cancer receiving antihormonal therapy with anti-HER2 treatment and recurrent vaginal vault prolapse who presented with acute vaginal evisceration of the small bowel. She had undergone a vaginal hysterectomy for pelvic organ prolapse 3 years earlier. Emergency surgical management was performed via laparotomy with repositioning of the small bowel, vaginal cuff closure, and concomitant sacrocolpopexy. One week later, re-laparotomy was required due to an open abdomen associated with paralytic small bowel ileus; bowel resection was not necessary. The last follow-up was carried out 21 months postoperatively. The patient was asymptomatic and showed no evidence of recurrent prolapse.

**Conclusions:**

This case highlights a rare late-onset VCDE in a patient receiving combined antihormonal and anti-HER2 therapy, a clinical context not previously described. While causality cannot be established, this report raises the hypothesis of a potential association between targeted therapy and impaired tissue integrity.

## Background

Vaginal cuff dehiscence and evisceration (VCDE) after hysterectomy is a rare but potentially serious postoperative complication. The likelihood of VCDE varies by surgical approach. Evidence from large cohort studies and meta-analyses indicates that the highest incidence is observed after total laparoscopic hysterectomy, followed by robotic-assisted hysterectomy. In contrast, vaginal and abdominal hysterectomy are associated with substantially lower rates. Reported incidences range from approximately 0.64% to 1.35% following total laparoscopic hysterectomy, around 1.64% after robotic hysterectomy, and between 0.02% and 0.21% after vaginal or abdominal procedures. The increased risk with TLH and robotic techniques is attributed to factors such as the use of thermal energy during colpotomy and differences in cuff closure technique [1–3]. However, direct comparison of incidence estimates across studies is limited by heterogeneity in study design (predominantly retrospective cohorts), varying definitions of VCDE, differences in follow-up duration, and incomplete reporting of cuff closure technique (laparoscopic vs transvaginal). These methodological differences should be considered when interpreting route-specific incidence rates. The interval between initial hysterectomy and VCDE has been described as up to 30 years. Risk factors such as age, vaginal atrophy, poor wound healing, chronic diseases, infections, and hematoma are discussed [4].

We present the case of a patient with VCDE 3 years after a vaginal hysterectomy, who was under anti-hormonal and anti-HER2 therapy due to hormone receptor-positive and HER2-positive breast cancer. Although vaginal atrophy is a recognised risk factor for VCDE, there is currently no evidence regarding a potential association between anti-HER2 therapy and the occurrence of this complication. This case report, therefore, adds to the limited literature on VCDE in patients receiving anti-HER2 therapy.

## Case presentation

**Fig. 1:**
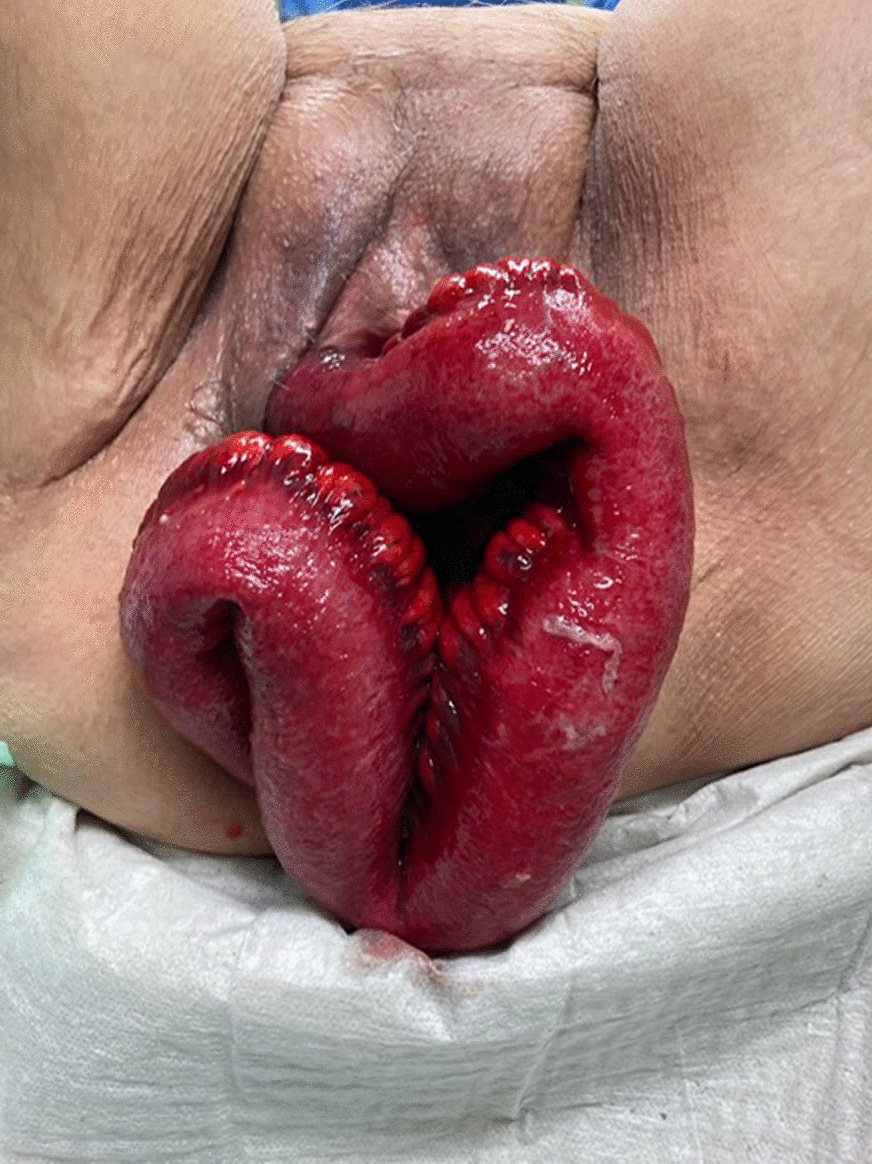
Preoperative view demonstrating complete vaginal cuff dehiscence with evisceration of edematous small-bowel loops through the vaginal introitus; the bowel appeared viable without macroscopic signs of ischemia

An 81-year-old White woman presented to the emergency department with acute lower abdominal pain and a sudden vaginal bulge that had started 3 h earlier without a precipitating event. Three years earlier, she had undergone vaginal hysterectomy with anterior colporrhaphy and tension-free transobturator tape insertion for pelvic organ prolapse and urinary stress incontinence. Five months before presentation, a recurrent vaginal vault prolapse (POP-Q stage III) had been diagnosed; conservative and surgical treatment options were deferred due to ongoing trastuzumab emtansine (T-DM1) and letrozole therapy for hormone receptor-positive, Her2-positive breast cancer which had been administered continuously for 5 months (letrozole) and 3 months (T-DM1) prior to presentation, with ongoing exposure up to the acute event. Clinical examination revealed complete vaginal cuff dehiscence with evisceration of edematous but viable small bowel (Fig. 1). Laboratory investigations showed a white blood cell count (WBC) of 10.75 G/L and a C-reactive protein (CRP) level of 5.76 mg/l, with otherwise normal parameters. Emergency laparotomy was indicated, and intravenous antibiotics with cefuroxime (1.5 g three times daily) and metronidazole (500 mg twice daily) were started. Broad-spectrum antibiotic therapy was initiated empirically to cover potential translocation of intestinal bacteria and reduce the risk of peritonitis. Letrozole was temporarily withheld.

**Fig. 2:**
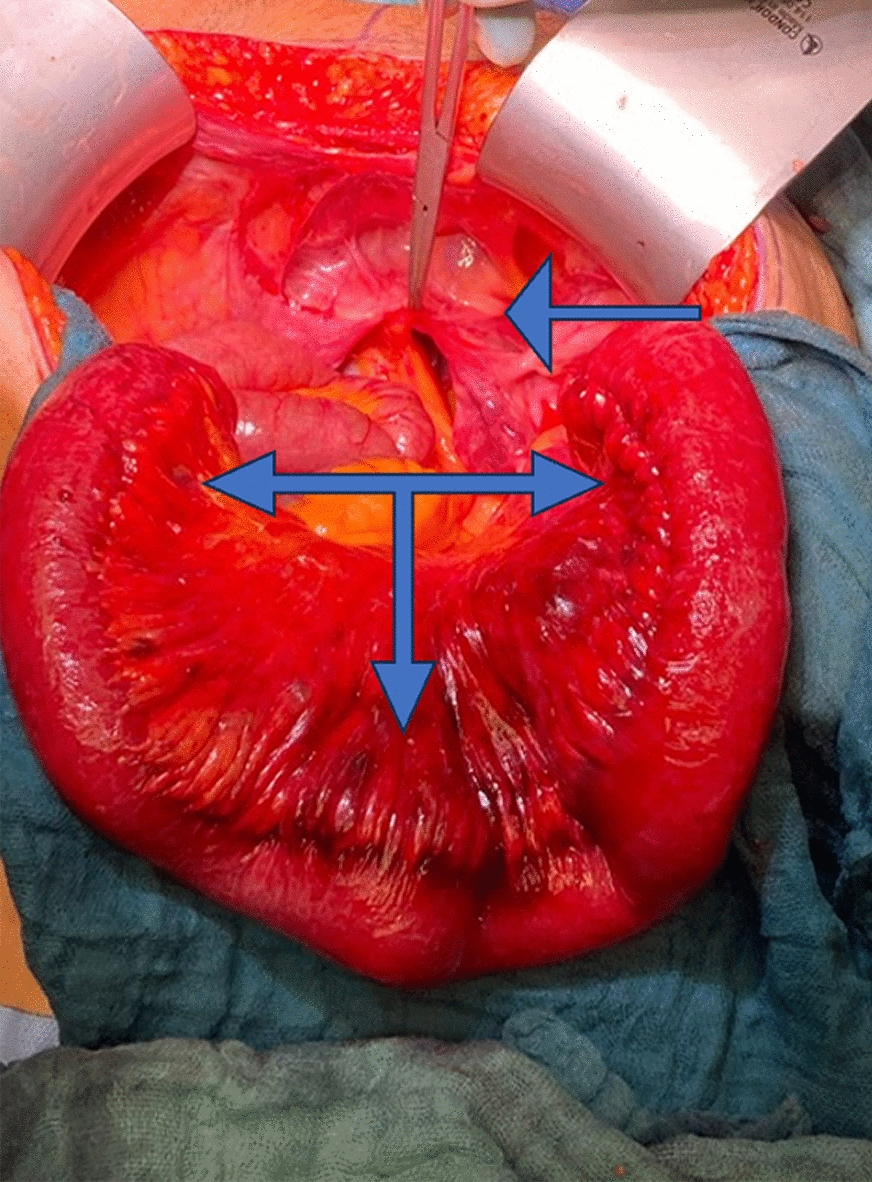
Intraoperative view after gentle reduction of the small bowel into the abdominal cavity, illustrating restored bowel positioning and preserved perfusion prior (three-piece arrow) to vaginal cuff closure (single arrow)

The acute presentation, with visible transvaginal protrusion of small bowel and abdominal pain, made VCDE clinically evident. In this context, the differential diagnoses are limited. Conditions such as vaginal vault prolapse or an enterocele without rupture may present with a vaginal bulge but can be readily distinguished by intact vaginal epithelium and the absence of exposed bowel. Clinical examination demonstrated full-thickness cuff separation with visible small-bowel loops, confirming the diagnosis. In contrast to many acute abdominal conditions, the diagnosis in this case was primarily clinical and did not require additional imaging. The absence of peritoneal signs and only mildly elevated inflammatory markers suggested preserved bowel viability, which was subsequently confirmed intraoperatively. Given the extent of bowel evisceration and uncertainty regarding bowel viability, an abdominal approach was preferred to allow full inspection of the intestine and safe management if resection had been required. During the 113-min operation, the small bowel was carefully repositioned into the abdominal cavity after minimal dilation of the vaginal vault. The vaginal cuff margins were deepithelialised, biopsied, and closed with a continuous PDS-0 suture. A concomitant sacrocolpopexy was performed for vaginal vault prolapse repair. The decision to perform concomitant sacrocolpopexy was based on recurrent vault prolapse, a key mechanical risk factor for VCDE recurrence. Given the pre-existing recurrent vault prolapse, definitive apical support was considered important to reduce mechanical strain on the newly closed cuff and to minimise the risk of early recurrence. A staged approach was weighed against the patient’s age and the prospect of a second anesthetic exposure. We therefore opted for concomitant sacrocolpopexy, accepting a slightly longer operative time in exchange for immediate restoration of apical support. Intraoperative surgical consultation confirmed bowel viability, and bowel resection was not required as edema and venous congestion resolved (Fig. 2). Fascial closure was achieved with continuous PDS sutures, and abdominal drains were placed. Postoperatively, the patient received prophylactic anticoagulation, continued intravenous antibiotic therapy with cefuroxime and metronidazole, and bowel-regulating measures (including early oral nutrition, laxatives, mobilisation, and close postoperative monitoring). Low-dose aspirin (100 mg once daily) was initiated on postoperative day 1 to support bowel perfusion. Histopathological examination of vaginal cuff biopsies revealed no evidence of malignancy.

On postoperative day 7, the patient developed acute wound dehiscence with complete fascial separation (burst abdomen), prompting urgent re-laparotomy and wound revision. Intraoperatively, there were no signs of intestinal perforation, intra-abdominal infection, or anastomotic pathology. A paralytic small-bowel ileus with abdominal distension was noted and considered a contributing factor due to increased intra-abdominal pressure. Fascial closure was performed using PDS retention sutures with additional lateral reinforcement. Postoperatively, the patient passed stools regularly while receiving bowel-regulating medication as described above. Because of rising inflammatory parameters (CRP 185 mg/l, WBC 7,48 G/l), the antibiotic treatment was changed to vancomycin (1000 mg twice daily) with metronidazole on the 13th postoperative day. At that time, blood cultures and wound swabs were obtained. Given persistently elevated inflammatory markers and ongoing wound secretion, antibiotic therapy was escalated to include vancomycin to ensure coverage of gram-positive pathogens, including resistant organisms, according to institutional protocol. The wound continued to secrete, so a computed tomography scan was performed. A recurrent open abdomen was ruled out, and only a slight superficial skin dehiscence was found. Regular wound lavages were carried out daily, resulting in secondary wound closure. The patient received two red blood cell concentrates in the course of her anemia (hemoglobin 8.7 g/dl and 8.4 g/dl). On the 28th postoperative day, the patient could be discharged. The patient was specifically counseled to seek immediate medical attention for any sudden vaginal bulge or tissue protrusion, acute pelvic/abdominal pain, or any other unexpected symptoms. Letrozole was continued, as was treatment with T-DM1 seven weeks postoperatively.

The last follow-up was at 21 months postoperatively, and the patient was symptom-free, with a POP-Q stage 0 (pelvic organ prolapse quantification, POP-Q point C = −7).

## Discussion

VCDE after a hysterectomy is a rare complication. Its exact incidence is still unknown, ranging from 0.032% to 1.2%. [4] To our knowledge, this is the first report describing VCDE occurring during active anti-HER2 therapy in combination with antihormonal treatment. Ginsberg et al. describe a common triad of VCDE: lack of estrogen, prior vaginal hysterectomy, and presence of an enterocele [5]. Some studies describe a higher rate of VCDE after laparoscopic hysterectomy compared to a vaginal or abdominal approach. After a robotic-assisted hysterectomy, VCDE seems even higher than after a conventional laparoscopic hysterectomy [1, 2]. However, there is not always a distinction in these studies whether cuff closure was performed laparoscopically or vaginally. Interestingly, Uccella et al. estimated that a randomised trial would require 2617 women per arm to detect a significant difference in VCDE rates between laparoscopic and transvaginal cuff closure techniques [1]. There are two main explanations for a possible lower rate of VCDE after a vaginal and abdominal approach: thermal damage and tension on the knots and sutures. First, thermal damage might weaken the tissue during laparoscopic colpotomy [6, 7]. That is why Uccella et al. suggest performing a supracervical hysterectomy instead of a total hysterectomy when possible [1]. Second, regarding the suture technique, the tension on the knots and suture might be higher when vaginal cuff closure is performed using the hands [1, 8]. This might also explain why VCDE seems more frequent after a robotic vaginal cuff closure due to the missing haptic feeling [7].

The indication for hysterectomy is also associated with VCDE risk, as patients who underwent hysterectomy for malignant disease had a higher risk of VCDE than those with a benign indication. The reason, therefore, might be treatment with chemotherapy and radiation, leading to impaired tissue healing and vaginal atrophy [1]. Although our patient underwent hysterectomy for a benign indication (prolapse), additional risk factors were present: recurrent prolapse and vaginal atrophy due to her breast cancer and antihormonal and anti-HER2 therapy. There is no data suggesting that the mitosis inhibitor mertansine in T-DM1 impairs wound healing. Although clinical evidence is lacking, a theoretical biological plausibility may be considered. HER2/ErbB signaling contributes to epithelial homeostasis and wound repair, and its inhibition could hypothetically affect mucosal integrity. However, this remains speculative and is not supported by clinical evidence. In addition, antibody–drug conjugates such as T-DM1 deliver a microtubule inhibitor to HER2-expressing cells, potentially delaying cellular turnover in regenerating tissue. These considerations remain speculative and hypothesis-generating. Conservative treatment options (pessary) and surgical therapy were offered to the patient. However, the patient chose a wait-and-see strategy.

Our patient presented at our clinic with acute abdominal pain; there was no precipitating event, as spontaneous VCDE can be seen in up to 70% of patients [9]. Furthermore, VCDE is described after a period of up to 30 years after hysterectomy as a late-onset complication. Eoh et al. describe a later onset in postmenopausal women compared to premenopausal patients. The cut-off for an early onset, however, was set 8 weeks postoperatively. After a minimally invasive hysterectomy, the onset is also described earlier than after a total abdominal hysterectomy [10].

Some authors conclude that a yearly consultation after a hysterectomy might prevent some cases of VCDE [9]. We suggest a pragmatic, risk-adapted approach for postmenopausal patients on combined antihormonal and anti-HER2 therapy with concomitant prolapse or symptomatic atrophy: a pelvic examination at baseline (therapy initiation), again after 3–6 months, and thereafter annually, with earlier assessment if symptoms occur. However, as shown in our patient, even close surveillance cannot prevent all cases of VCDE. Other authors state that, instead of close surveillance, the anatomic aspects after vaginal hysterectomy should be the focus. Vaginal hysterectomy, especially in combination with anterior vaginal wall repair, can lead to a shortening of the vagina and to a vaginal apex which is abnormally unsupported over the urogenital hiatus. This changes the pressure transmission, resulting in a higher pressure of the vaginal apex. Instead of close surveillance after hysterectomy, Khunda and Jones suggest improving the awareness of these changes after vaginal hysterectomy [11].

Currently, there is no consensus on the ideal method of surgical repair of VCDE. More than half of the reported cases are performed vaginally (51%), about 31% through an abdominal approach, and sometimes a combined procedure is done. Although there is no consensus, the recurrence of VCDE is only 4% [4]. Relevant risk factors for recurrence after surgical treatment of VCDE are suboptimal surgical technique during cuff closure, postoperative infection, early mechanical stress such as sexual intercourse, adjuvant therapies (chemotherapy or brachytherapy), and a low body mass index [7, 12, 13]. To reduce the risk of recurrence after surgical treatment of a VCDE, current literature recommends, in particular, careful surgical technique with sufficient tissue quantities, the use of barbed sutures, and advises avoiding premature mechanical stress and monitoring postoperative infections. These measures have been shown to optimise healing and significantly reduce the risk of recurrence [13–15].

Another important aspect of VCDE is the need for bowel resection. Our case report shows that bowel resection is not always necessary, even with prominent swelling and low bowel movement. A bowel resection carries high morbidity and mortality, especially in the elderly, and should be performed only when necessary. It can also be performed vaginally when VCDE is present [16]. A two-stage procedure may therefore be an option to avoid a primary bowel resection, even if it means re-laparotomy, as it was necessary in this case. Intestinal regulation measures to prevent ileus, such as adequate fluid intake, early mobilisation, and a normal diet, can prevent the development of ileus. In the case presented here, the patient underwent another operation for an open abdomen and mild ileus symptoms without bowel resection. Postoperative ileus has been described as a contributor to increased intra-abdominal pressure, which may compromise fascial integrity and predispose to wound dehiscence. In the present case, we consider the burst abdomen most likely multifactorial, with ileus-related distension as a plausible contributing mechanism rather than a proven cause.

This case describes a previously undescribed clinical constellation and may serve as a signal-generating observation for future research, highlighting a rare late-onset presentation three years after vaginal hysterectomy in a patient receiving combined antihormonal and anti-HER2 therapy. Although no causal association can be established, the case highlights the importance of considering potential risk constellations affecting tissue integrity in postmenopausal patients. This observation is hypothesis-generating and must not be interpreted as evidence of causality. VCDE is multifactorial, and established risk factors in this patient—advanced age, postmenopausal atrophy, and recurrent vault prolapse with altered apical support—provide plausible alternative explanations. Nevertheless, documenting VCDE occurring during anti-HER2 exposure may help identify a potential clinical signal and guide future systematic studies. Furthermore, it demonstrates that bowel evisceration does not necessarily require bowel resection, thereby supporting an individualised, bowel-preserving surgical approach, particularly in elderly patients.

The limitation of this report is its single-case design, which precludes establishing causality between anti-HER2 therapy and VCDE. Mechanistic evidence linking T-DM1 or letrozole to impaired tissue healing is lacking. In addition, alternative explanations based on established risk factors are plausible. Any potential association between anti-HER2 therapy and VCDE must therefore be interpreted with caution and cannot be inferred from a single case.

## Conclusion

This case highlights a rare late-onset VCDE in a postmenopausal patient with recurrent vault prolapse who is receiving combined antihormonal and anti-HER2 therapy—a clinical constellation for which evidence remains limited and causal inference is not possible. The report is hypothesis-generating and may inform future systematic evaluation of potential risk constellations affecting tissue integrity. In addition, it illustrates that bowel evisceration does not necessarily require bowel resection, supporting an individualised, bowel-preserving surgical approach when bowel viability is confirmed.

## Data Availability

The dataset and materials used in this study are available from the corresponding author upon reasonable request.

## Abbreviations

VCDE: Vaginal cuff dehiscence (and evisceration)
POP-Q: Pelvic organ prolapse quantification
T-DM1: Trastuzumab emtansine
WBC: White blood cell count
CRP: C-reactive protein

## References

[1] Uccella S, Bogani G, Ghezzi F. Vaginal cuff dehiscence after laparoscopic and robotic hysterectomy: is endoscopic colporrhaphy a waste of time? Am J Obstet Gynecol. 2012;206(3):e10–1. 10.1016/j.ajog.2011.10.861. 22118963 10.1016/j.ajog.2011.10.861

[2] Hur HC, Donnellan N, Mansuria S, Barber RE, Guido R, Lee T. Vaginal cuff dehiscence after different modes of hysterectomy. Obstet Gynecol. 2011;118(4):794–801. 10.1097/AOG.0b013e31822f1c92. 21934442 10.1097/AOG.0b013e31822f1c92

[3] Ikki A, Aoki Y, Kanno M, Tsumura S, Fusegi A, Abe A, Netsu S, Omi M, Tanigawa T, Okamoto S, Nomura H, Kanao H. Comparison of the incidence of vaginal cuff dehiscence by hysterectomy route and type based on experienced surgeons’ outcome. Int J Gynaecol Obstet. 2025;170(1):222–32. 10.1002/ijgo.16189. 39891484 10.1002/ijgo.16189

[4] Cronin B, Sung VW, Matteson KA. Vaginal cuff dehiscence: risk factors and management. Am J Obstet Gynecol. 2012;206(4):284–8. 10.1016/j.ajog.2011.08.026. 21974989 10.1016/j.ajog.2011.08.026 PMC3319233

[5] Ginsberg DA, Rovner ES, Raz S. Vaginal evisceration. Urology. 1998;51(1):128–9. 9457306 10.1016/s0090-4295(97)00477-9

[6] Hur HC, Guido RS, Mansuria SM, Hacker MR, Sanfilippo JS, Lee TT. Incidence and patient characteristics of vaginal cuff dehiscence after different modes of hysterectomies. J Minim Invasive Gynecol. 2007;14(3):311–7. 10.1016/j.jmig.2006.11.005. 17478361 10.1016/j.jmig.2006.11.005

[7] Pinho G, Liu YX, Kim S, Lian X. Prevention and management of vaginal cuff dehiscence: an updated review. Curr Opin Obstet Gynecol. 2022;34(4):250–5. 10.1097/GCO.0000000000000791. 35895968 10.1097/GCO.0000000000000791

[8] Muffly T, McCormick TC, Dean J, Bonham A, Hill RFC. An evaluation of knot integrity when tied robotically and conventionally. Am J Obstet Gynecol. 2009;200(5):e18-20. 10.1016/j.ajog.2008.08.058. 19111718 10.1016/j.ajog.2008.08.058

[9] Croak AJ, Gebhart JB, Klingele CJ, Schroeder G, Lee RA, Podratz KC. Characteristics of patients with vaginal rupture and evisceration. Obstet Gynecol. 2004;103(3):572–6. 10.1097/01.AOG.0000115507.26155.45. 14990423 10.1097/01.AOG.0000115507.26155.45

[10] Eoh KJ, Lee YJ, Nam EJ, Jung HI, Kim YT. Vaginal cuff dehiscence and a guideline to determine treatment strategy. J Pers Med. 2023;13(6):890. 10.3390/jpm13060890. 37373878 10.3390/jpm13060890 PMC10303730

[11] Khunda A, Jones D. Reply. Am J Obstet Gynecol. 2006;194(6):1744. 10.1016/j.ajog.2005.10.219. 16731099 10.1016/j.ajog.2005.10.221

[12] Drudi L, Press JZ, Lau S, Gotlieb R, How J, Eniu I, Drummond N, Brin S, Deland C, Gotlieb WH. Vaginal vault dehiscence after robotic hysterectomy for gynecologic cancers: search for risk factors and literature review. Int J Gynecol Cancer. 2013;23(5):943–50. 10.1097/IGC.0b013e31828f38e1. 23669442 10.1097/IGC.0b013e31828f38e1

[13] Nezhat C, Kennedy Burns M, Wood M, Nezhat C, Nezhat A, Nezhat F. Vaginal cuff dehiscence and evisceration: a review. Obstet Gynecol. 2018;132(4):972–85. 10.1097/AOG.0000000000002852. 30204700 10.1097/AOG.0000000000002852

[14] Uccella S, Zorzato PC, Kho RM. Incidence and prevention of vaginal cuff dehiscence after laparoscopic and robotic hysterectomy: a systematic review and meta-analysis. J Minim Invasive Gynecol. 2021;28(3):710–20. 10.1016/j.jmig.2020.12.016. 33348012 10.1016/j.jmig.2020.12.016

[15] Uccella S, Malzoni M, Cromi A, Seracchioli R, Ciravolo G, Fanfani F, Shakir F, Gueli Alletti S, Legge F, Berretta R, Corrado G, Casarella L, Donarini P, Zanello M, Perrone E, Gisone B, Vizza E, Scambia G, Ghezzi F. Laparoscopic vs transvaginal cuff closure after total laparoscopic hysterectomy: a randomized trial by the Italian Society of Gynecologic Endoscopy. Am J Obstet Gynecol. 2018;218(5):500.e1-500.e13. 10.1016/j.ajog.2018.01.029. 29410107 10.1016/j.ajog.2018.01.029

[16] Moen MD, Desai M, Sulkowski R. Vaginal evisceration managed by transvaginal bowel resection and vaginal repair. Int Urogynecol J Pelvic Floor Dysfunct. 2003;14(3):218–20. 10.1007/s00192-003-1056-1. 12955347 10.1007/s00192-003-1056-1

